# The Association Between Medication Literacy and Blood Pressure Control in Patients With Hypertension in a Multi-ethnic County in China

**DOI:** 10.7759/cureus.108707

**Published:** 2026-05-12

**Authors:** Zheng Feng, Liao Liqin, Xiang Defen, Xiang Fang, Lu Jing

**Affiliations:** 1 Nursing Department, Department of Cardiovascular Medicine, The Third Xiangya Hospital, Central South University, Changsha, CHN; 2 Department of Cardiovascular Medicine, Huayuan People's Hospital, Huayuan Town, CHN

**Keywords:** hypertension, medication literacy, multi-ethnic, self-management, social support

## Abstract

Background and objective

Hypertension is a significant risk factor for cardiovascular disease. Patients’ medication literacy plays a key role in managing hypertension. This study sought to assess the level and determinants of medication literacy and blood pressure (BP) control among patients with primary hypertension and to examine the relationship between medication literacy and BP control.

Methods

This cross-sectional study was conducted in Huayuan County, Hunan Province, China. Outpatients aged 18-85 years who had been diagnosed with hypertension and visited the outpatient department were included as study participants. Participants were recruited using a convenience sampling technique. Medication literacy was assessed using the Revised Chinese Medication Literacy Scale for Hypertensive Patients (C-MLSHP-R), and factors associated with poor medication literacy among hypertensive patients were examined, including socioeconomic and demographic data, the Self-Maintenance Scale for Hypertension Patients (SMSPH), and the Social Support Rating Scale (SSRS). In addition, patients’ blood pressure control status was monitored.

Results

A total of 642 questionnaires were collected, with an average medication literacy score of (28.18 ± 11.40), indicating a moderate level. Among the patients, 46.73% of participants achieved optimal BP control. Univariate analysis revealed statistically significant differences in medication literacy across subgroups defined by age, education level, primary income source, health insurance type, perceived financial status, having a medical background, having cohabiting family members with a medical background, having cohabiting family members with hypertension, total self-management score, and social support level (p < 0.05). Pearson correlation analysis demonstrated a significant positive correlation between medication literacy and self-management level (r = 0.531, p < 0.01). Multiple linear regression analysis identified education level, insurance type, having a medical background, having cohabiting family members with a medical background, total self-management score, and social support level as significant predictors of medication literacy, collectively explaining 48.6% of the variance (F = 21.164, p < 0.01). Chi-square test results demonstrated that higher education levels were associated with better BP control compliance (χ2 = 11.639, p = 0.020). The SMSPH total score, C-MLSHP-R total score, and medication knowledge score were significantly associated with BP control (p-values: 0.044, 0.030, and 0.021, respectively). Binary logistic regression analysis indicated that medication literacy (odds ratio (OR): 1.039, 95% confidence interval (CI): 1.006 to 1.073) was a significant contributor to BP control compliance (Wald χ2 = 5.327, p = 0.021).

Conclusions

The medication literacy level of patients with hypertension in Huayuan County requires improvement. Medication literacy is a key factor in improving BP control. Targeted attention is warranted for patients with lower education levels, without medical insurance, without a medical background, with poor self-management ability, and with limited social support.

## Introduction

According to the World Health Organization Global Report on Hypertension 2025, the global prevalence of hypertension among adults aged 30 to 79 years reached 33% in 2024, affecting an estimated 1.4 billion people worldwide [1]. However, blood pressure (BP) control remains challenging in clinical and public health settings. Among those diagnosed with hypertension, the majority (> 85%) receive treatment, but only approximately one quarter achieve adequate control [2]. The prevalence of hypertension among adults aged 18 and above in China is 27.5%, and the estimated number of patients has reached 245 million. Nevertheless, there remains substantial room for improvement in the treatment rate (45.8%) and control rate (16.8%) [3]. In the absence of appropriate medical supervision, self-medication is a common phenomenon among patients with hypertension [4]. Effective medication management is a critical skill for patients with hypertension. Medication literacy has been identified as one of the predictors of medication self-management behavior [5].

Medication literacy refers to the knowledge, skills, and confidence that enable patients to access, comprehend, assess, and use medication information, which supports rational medication-related decisions and behaviors [6]. Medication literacy plays an essential role in patients’ self-management of medications. Evidence shows that low medication literacy is significantly associated with poor medication adherence and inadequate BP control [7,8]. Poor medication adherence results in greater cumulative exposure to elevated BP [9,10]. Furthermore, social support is an important factor in hypertension management. Research by Shen et al. demonstrates that social support is positively associated with medication literacy in older Chinese patients living with hypertension [11].

Currently, attention to medication literacy among Chinese patients with hypertension is largely focused on urban or economically developed areas. Studies examining medication literacy and BP management among individuals with hypertension in remote mountainous and multi ethnic rural regions remain limited. Huayuan County lies in the northwest of Hunan Province, within the Wuling Mountain Area, and is recognized as a significant birthplace and cultural hub of the Miao ethnic group. The villages are sparsely distributed, which makes access to healthcare services difficult. The local population strongly favors pickled and fermented foods, which pose a considerable risk for cardiovascular disease. A survey conducted in the ethnic minority areas of western Hunan reported that the prevalence of hypertension is 52.34% [11,12], which is higher than the national prevalence among adults of 27.5% [3]. This study aims to investigate the status of medication literacy and BP control in patients with hypertension in Huayuan County, a multi ethnic county of China, and to examine the relationship between medication literacy and BP control.

## Materials and methods

Study design and sampling

Patients were recruited using convenience sampling from the hypertension outpatient clinic of Huayuan County People’s Hospital between August 1, 2024, and August 30, 2025. The sample size was estimated using Cochran’s formula for a cross-sectional study design. The following statistical formula was employed:

$n = \frac{Z^2 P(1 - P)}{E^2}$

The overall BP control rate was 16.8% [3]. The sample size calculation assumed a 5% margin of error and a 95% confidence interval (CI). To account for potential nonresponse and incomplete questionnaires, an additional 10% was added to the final sample size estimate. The calculated sample size was 239. Finally, a total of 642 participants were successfully recruited and completed the questionnaire.

Participant recruitment

The inclusion criteria were as follows: (1) age between 18 and 85 years, and the absence of language communication barriers; (2) diagnosis of hypertension according to the China Hypertension Prevention and Treatment Guidelines (2024 Revised Edition) [12], that is, systolic blood pressure ≥ 140 mmHg and/or diastolic blood pressure ≥ 90 mmHg; (3) use of at least one antihypertensive medication for a minimum of two weeks [13]. The exclusion criteria were as follows: (1) patients with mental disorders; (2) terminal patients or those with conditions that limit adherence to medical advice, such as end-stage cancer or active severe mental illness; (3) patients who were currently participating in, or had participated in, hypertension-related intervention programs within the past 30 days.

The patients completed the questionnaire anonymously after signing informed consent. The survey was administered through face-to-face interviews. For older adults, individuals with limited literacy, or patients with visual impairment, investigators provided detailed explanations and assisted in completing each questionnaire and each scale item. After completion, data were collected immediately, and any missing information was checked on site. Patients were promptly asked to complete any missing responses. A total of 690 questionnaires were distributed, and 642 valid questionnaires were recovered after excluding invalid responses, with a response rate of 93%.

Measurements

The questionnaire consisted of four main sections. The first section assessed participants’ demographic and socioeconomic characteristics, including sex, age, ethnicity, education level, marital status, type of medical insurance, primary source of income, financial status, and presence of a medical background. According to the classification criteria by Luo Chun [14], age was categorized into four groups: youth (18-39 years), prime age (40-59 years), younger elderly (60-79 years), and oldest old (80-85 years).

The second section aimed to assess patients’ medication literacy. The C-MLSHP-R was used to measure medication literacy. It consists of four domains: knowledge, attitude, skills, and behavior, with a total of 18 items. The medication knowledge dimension includes four items, all of which are multiple-choice questions, with 1 point awarded for selecting each correct option. The medication attitude dimension includes three items. The medication behavior dimension includes four items, all scored on a 5-point Likert scale (0 to 4), and items A1 to A3 in the medication attitude dimension are reverse-scored. The medication skills dimension contains seven items, with 1 point awarded for correct answers and 0 points for incorrect answers or selecting “I do not know”. The total score ranges from 0 to 51, with higher scores indicating higher levels of medication literacy. The Cronbach’s alpha coefficient of the scale is 0.802, and the split-half reliability coefficient is 0.709 [15]. The scale demonstrates good reliability and validity. In this study, the Cronbach’s alpha coefficient was 0.924.

The third section aimed to assess patients’ self-maintenance. The Self-Maintenance Scale for Hypertension Patients (SMSPH) is a 5-point Likert scale designed to assess self-management behaviors in hypertensive patients. It comprises 21 items across four dimensions: treatment management (eight items), diet and exercise management (five items), lifestyle management (five items), and risk factor management (three items). The scale includes 15 positive items and six reverse-scored items, with a total score range of 21 to 105. Higher scores indicate better self-management. The Cronbach's alpha coefficient of the scale is 0.854, and its content validity is 0.976 [15,16].

The fourth part assessed patients’ social support. The Social Support Rating Scale (SSRS) was designed to evaluate an individual’s social relationships across three dimensions: objective support, subjective support, and utilization of social support. It includes a total of 10 items. The sum of items 2, 6, and 7 represents objective support, which refers to the actual support that patients can directly perceive. The sum of items 1, 3, 4, and 5 represents subjective support, that is, the emotional support perceived by the individual. The sum of items 8, 9, and 10 represents utilization of social support, which indicates how individuals make use of available social support. The total score is 66 points, with scores < 22 indicating a low level, 23-44 indicating a moderate level, and 45-66 indicating a high level. Both the total scale and its three subscales have Cronbach’s alpha coefficients above 0.82, and the internal consistency is 0.92, demonstrating good reliability and validity [17].

The BP control achievement rate refers to the percentage of patients with controlled BP among the total number of surveyed patients. When patients monitored their BP at home, BP control achievement was determined based on the BP measured during the most recent week of home monitoring. When patients were not monitoring their BP at home or could not provide accurate information, BP control achievement was assessed based on immediate BP measurements. An immediately-measured BP was obtained using the same certified and calibrated upper-arm electronic sphygmomanometer. When measuring BP, measurements were repeated at intervals of 30-60 seconds, and the average of two readings was recorded. If the difference between the two readings of systolic or diastolic pressure exceeded 10 mmHg, another measurement was taken, and the average of three readings should be recorded [12]. The BP control targets [12] in this study were defined as follows: (1) the general reference standard was 140/90 mmHg, with a tolerable target of < 130/80 mmHg; (2) for elderly patients aged ≥ 80 years, the recommended BP target was < 150/90 mmHg, and a tolerable target of < 140/90 mmHg was recommended.

Data analysis

The questionnaire data were independently entered by two data entry personnel, and the dataset was checked and cleaned after consistency verification. Data analysis was performed using SPSS Statistics 26.0 software (IBM Corp., Armonk, NY). Measurement data that followed a normal distribution were expressed as mean ± standard deviation (SD), while non normally distributed data were presented as median and interquartile range. Differences between two groups were analyzed using the independent samples t-test, and differences among multiple groups were assessed using one-way ANOVA. Categorical data were presented as frequencies and percentages. Group comparisons were conducted using the chi-square test, and bivariate relationships were assessed using Pearson correlation analysis. Single-factor analysis, Pearson correlation analysis, and multiple linear regression were used to identify factors influencing medication literacy in patients with primary hypertension. The chi-square test was used to examine factors associated with BP control. Binary logistic regression was applied to explore the association between medication literacy and BP control. The level of statistical significance was set at α = 0.05.

## Results

The mean age of the 642 patients with hypertension was 61.27 ± 12.64. There were 333 males (51.87%) and 309 females (48.13%). Among them, 72 were of Han nationality (11.21%), 411 were of Miao nationality (64.02%), and 156 were of Tujia nationality (24.30%). The C-MLSHP-R scores ranged from 2 to 51, with a mean score of 28.18 ± 11.40, indicating a moderate level of medication literacy. Univariate analysis revealed statistically significant differences in medication literacy according to the following factors: age, education level, income source, type of medical insurance, self-reported financial status, personal medical background, the presence of a medical background among cohabiting family members, cohabiting family members with hypertension, total SMSPH score, and level of social support (p < 0.05).

The study subjects were divided into the BP-controlled group (n = 300) and the BP-uncontrolled group (n = 342) according to whether BP targets were achieved. It was shown that there were statistically significant differences in the BP control rate only among patients with different age and education levels (p < 0.05). The baseline characteristics of study participants in the BP-controlled group and the BP-uncontrolled group are presented in Table 1.

**Table 1:** Baseline characteristics of study participants in the BP-controlled group and the BP-uncontrolled group (N = 642) ^*^Indicates p < 0.05. ^a^Indicates independent samples t-test. ^b^Indicates one-way ANOVA BP: blood pressure; C-MLSHP-R: the Revised Chinese Medication Literacy Scale for Hypertensive Patients; SD: standard deviation

Variables	Categories	BP-controlled group (n = 300), n (%)	BP-uncontrolled group (n = 342), n (%)	χ^2^	P	N (%)	The C-MLSHP-R scores, mean ± SD	t/F	P-value
Age, years	18-39	24 (8.0)	27 (7.9)	1.373	0.712	51 (7.9)	36.88 ± 8.77	4.423^b^	0.005
	40-59	99 (33.0)	93 (27.2)			192 (29.9)	28.63 ± 12.68		
	60-79	168 (56.0)	204 (59.6)			372 (57.9)	27.10 ± 10.45		
	80-85	9 (3.0)	18 (5.3)			27 (4.2)	23.44 ± 12.52		
Nationality	Han	42 (14.0)	30 (8.8)	2.950	0.399	72 (11.2)	31.46 ± 12.46	1.110^b^	0.346
	Miaos	180 (60.0)	231 (67.5)			411 (64.0)	27.88 ± 11.03		
	Tujia	75 (25.0)	81 (23.7)			156 (24.3)	27.25 ± 11.83		
	Others	3 (1.0)	0 (0)			3 (0.5)	39.00		
Gender	Male	144 (48.0)	189 (55.3)	1.126	0.289	333 (51.9)	29.47 ± 10.49	2.354^a^	0.087
	Female	156 (52.0)	153 (44.7)			309 (48.1)	26.80 ± 12.21		
Educational level (years)	＜6	90 (30.0)	123 (35.9)	11.639	0.020	213 (33.2)	22.75 ± 10.66	47.896^b^	＜0.001^*^
	6-9	57 (19.0)	105 (30.7)			162 (25.2)	26.59 ± 10.27		
	9-12	60 (20.09)	69 (20.2)			129 (20.1)	29.56 ± 9.33		
	12-15	54 (18.0)	30 (8.8)			84 (13.1)	35.29 ± 10.84		
	＞15	39 (13.0)	15 (4.4)			54 (8.4)	40.06 ± 11.40		
Marital status	Single	6 (2.0)	3 (0.9)	0.982	0.806	9 (1.4)	34.00 ± 8.19	2.817^b^	0.421
	Married	273 (91.0)	306 (89.5)			579 (90.2)	28.34 ± 11.56		
	Divorced	3 (1.0)	6 (1.7)			9 (1.4)	20.00 ± 10.54		
	Widowed	18 (6.0)	27 (7.9)			45 (7.0)	26.60 ± 9.77		
Medical insurance	None	3 (1.0)	6 (1.7)	0.433	0.805	9 (1.4)	22.33 ± 9.24	19.628^b^	＜0.001^*^
	New Rural Cooperative Medical Scheme (NRCMS)	147 (49.0)	177 (51.8)			324 (50.5)	24.88 ± 10.61		
	Urban Resident Basic Medical Insurance (URBMI)	150 (50.0)	159 (46.5)			309 (48.1)	31.82 ± 11.21		
Income source	Farmland	108 (36.0)	114 (33.3)	7.321	0.396	222 (34.6)	23.54 ± 10.25	36.379^b^	＜0.001^*^
	Business	12 (4.0)	21 (6.1)			33 (5.1)	28.91 ± 10.13		
	Social welfare	9 (3.0)	9 (2.6)			18 (2.8)	30.50 ± 7.18		
	Migrant work	6 (2.0)	18 (5.3)			24 (3.7)	24.25 ± 13.97		
	Office clerk	75 (25.0)	54 (15.8)			129 (20.1)	35.47 ± 11.05		
	Pension	63 (21.0)	75 (21.9)			138 (21.5)	30.87 ± 11.40		
	Child support	18 (6.0)	21 (6.2)			39 (6.1)	23.00 ± 9.41		
	Others	9 (3.0)	30 (8.8)			39 (6.1)	26.92 ± 6.93		
Self-reported financial status	Very dissatisfied	18 (6.0)	33 (9.6)	3.269	0.514	51 (8.0)	22.94 ± 11.52	3.679^b^	0.006
	Not quite satisfied	54 (18.0)	63 (18.4)			117 (18.2)	28.13 ± 11.29		
	General	141 (47.0)	177 (51.8)			318 (49.5)	26.96 ± 10.81		
	Quite satisfied	57 (19.0)	51 (14.9)			108 (16.8)	33.92 ± 11.53		
	Very satisfied	30 (10.0)	18 (5.3)			48 (7.5)	29.06 ± 11.48		
Whether the cohabiting family members have high BP	Yes	144 (48.0)	159 (46.5)	0.490	0.825	303 (47.2)	29.90 ± 11.19	-2.102^a^	0.037
	No	156 (52.0)	183 (53.5)			339 (52.8)	26.65 ± 11.42		
Medical background	Yes	60 (20.0)	33 (9.6)	4.607	0.320	93 (14.5)	38.26 ± 9.66	-5.701^a^	＜0.001^*^
	No	240 (80.0)	309 (90.4)			549 (85.5)	26.48 ± 10.79		
Medical background of cohabiting family members	Yes	96 (32.0)	147 (43.0)	2.931	0.980	243 (37.9)	32.32 ± 10.41	-4.312^a^	＜0.001^*^
	No	204 (68.0)	195 (57.0)			399 (62.1)	25.66 ± 11.28		
Social support level	Low	0 (0)	9 (2.6)	3.541	0.170	9 (1.4)	11.67 ± 1.53	119.247^b^	0.030
	Middle	207 (69.0)	249 (72.8)			456 (71.0)	25.75 ± 10.29		
	High	93 (31.0)	84 (24.6)			177 (27.6)	35.29 ± 10.99		

Table 2 presents a correlation analysis between C-MLSHP-R and SMSPH. It shows that medication literacy levels were positively correlated with self-management, treatment management, diet and exercise management, and lifestyle habits management (r = 0.531, 0.568, 0.389, and 0.420, respectively; p < 0.001).

**Table 2:** Correlation analysis between C-MLSHP-R and SMSPH ^*^Indicates p < 0.01 C-MLSHP-R: the Revised Chinese Medication Literacy Scale for Hypertensive Patients; SMSPH: Self-Maintenance Scale for Hypertension Patients; SD: standard deviation

Variable	Average score (χ² ± SD)	C-MLSHP-R
C-MLSHP-R	28.18 ± 11.40	1
SMSPH	60.52 ± 13.32	0.531^*^
SMSPH - treatment management	22.39 ± 5.28	0.568^*^
SMSPH- diet and exercise management	15.75 ± 4.93	0.389^*^
SMSPH - self-management, treatment management	16.63 ± 4.51	0.420^*^
SMSPH - lifestyle habits management	5.75 ± 2.59	0.100

Taking medication literacy scores as the dependent variable, and age, education level, primary income source, type of medical insurance, perceived financial status, presence of medical background, presence of medical background among cohabiting family members, presence of hypertension among cohabiting family members, total self-management score, and level of social support as independent variables, a stepwise method was used to introduce multiple linear equations (α entry = 0.05, α exit = 0.10) for multivariate analysis. Ultimately, six variables were included in the regression equation, and the fitted equation was: Y = - 9.128 + 1.395(X1) + 2.845(X2) + 5.241(X3) + 4.307(X4) + 0.286(X5) + 4.771(X6). The fitted regression equation was statistically significant (F = 21.164, p < 0.01), with a multiple correlation coefficient (R) of 0.714 and a coefficient of determination (R²) of 0.510. This indicates that the proportion of medication literacy levels in patients with essential hypertension explained by education level, type of medical insurance, personal medical background, medical background of cohabiting family members, self-management level, and social support level was 48.6% (Table 3).

**Table 3:** Multivariate linear regression analysis of the influencing factors of medication literacy in patients with hypertension (N = 642) ^*^Indicates p＜0.01 R = 0.714; R^2^ = 0.510; adjusted R^2^ = 0.486 X1: Primary school or below = 0, junior high school = 1, senior high school or vocational school = 2, college = 3, bachelor's degree or above = 4. X2: Self-paid = 0, rural cooperative medical care = 1, urban resident medical insurance (including employee medical insurance) = 2. X3/X4: None = 0, available = 1. X6: Low = 0, medium = 1, high = 2. X5: Categorized as measurement data

	B	Standard error	β	t	P	F
Constant	-9.128	4.261		-2.142	0.033	21.164^*^
Education (X1)	1.395	0.641	0.58	2.176	0.031	
Medical insurance (X2)	2.845	1.363	0.132	2.088	0.038	
Medical background (X3)	5.241	1.820	0.162	2.879	0.004	
Whether the cohabiting family members have a medical background (X4)	4.307	1.193	0.184	3.611	＜0.001^*^	
Total score of SMSPH (X5)	0.286	0.051	0.334	5.662	＜0.001^*^	
Social support level (X6)	4.771	1.384	0.197	3.447	＜0.001^*^	

Table 4 presents a comparison of medication literacy and self-management levels among hypertensive patients in different BP control groups. When conducting the two-sample t-test, the total self-management score, treatment management dimension, diet and exercise management dimension, total medication literacy score, and medication knowledge dimension in the BP-controlled group were found to be higher than those in the BP-uncontrolled group (p < 0.001).

**Table 4:** Comparison of medication literacy and self-management level between hypertensive patients in different BP control groups (N = 642) ^*^Indicates p < 0.05 C-MLSHP-R: the Revised Chinese Medication Literacy Scale for Hypertensive Patients; SMSPH: Self-Maintenance Scale for Hypertension Patients; SD: standard deviation

Variables	BP-controlled group (n = 300), mean ± SD	BP-uncontrolled group (n = 342), mean ± SD	t	P
Medication knowledge	8.09 ± 5.83	5.51 ± 4.68	13.893	＜0.001^*^
Medication attitude	12.10 ± 4.80	10.02 ± 4.25	1.252	0.264
Medication skills	4.17 ± 2.41	2.95 ± 2.38	0.073	0.787
Medication behavior	11.11 ± 4.37	9.92 ± 3.89	1.550	0.215
Overall score for C-MLSHP-R	31.3 ± 12.17	25.45 ± 9.96	8.864	0.030
Treatment management	23.33 ± 5.09	21.56 ± 5.32	-2.476	0.007
Diet and exercise management	16.48 ± 4.95	15.11 ± 4.85	-2.051	0.021
Lifestyle management	17.17 ± 4.50	16.16 ± 4.49	-1.645	0.051
Risk factor management	5.49 ± 2.48	5.98 ± 2.66	-1.393	0.083
Overall score for SMSPH	62.47 ± 13.45	58.81 ± 13.03	-2.022	0.044

Binary logistic regression was employed to analyze the factors influencing BP control rates in hypertensive patients. Education level, the overall score of SMSPH, medication literacy, treatment management, diet and exercise management, and the total medication literacy score were selected as independent variables, while BP control status served as the dependent variable. The analysis followed a forward stepwise likelihood ratio method with α entry = 0.05, α exit = 0.10. The results indicated that medication literacy is the only factor influencing BP control among hypertensive patients. For each 1-point increase in the medication literacy score, the likelihood of BP control in these patients increased by 1.039 times (Table 5).

**Table 5:** Binary logistic regression of blood pressure control in primary hypertension patients (n = 642) C-MLSHP-R: the Revised Chinese Medication Literacy Scale for Hypertensive Patients; β: regression coefficient; χ2: chi-square test; OR: odds ratio; CI: confidence interval

Items	β	Wald χ^2^	OR	95% CI	P
Constant	-1.273	3.353	0.28	-	
C-MLSHP-R	0.38	5.327	1.039	1.006-1.073	0.021

## Discussion

Our findings showed that the average medication literacy score of patients with hypertension in Huayuan County was 28.18 ± 11.40, indicating a moderate level of medication literacy. This result is slightly lower than the findings of Qin et al. [13], suggesting a general deficiency in patients’ self-management ability regarding medication. This disparity in medication literacy may be attributed to differences in regional and population characteristics. Qin et al. investigated Han Chinese patients with hypertension in the Changsha area, while our study focused on multi-ethnic patients in remote regions of Hunan Province, China. However, our results showed no statistically significant differences in medication literacy scores across ethnic groups. This may be because the study was conducted at an urban hospital that mainly serves local residents. Although ethnic minorities were included, they were highly Sinicized, whereas those in remote mountainous areas seldom seek care in urban hospitals and were therefore underrepresented.

The factors influencing medication literacy include education level, type of medical insurance, personal medical history, medical history of cohabiting family members, self-management ability, and level of social support. The average medication literacy score among patients with fewer than six years of education (22.75 ± 10.66) was significantly lower than that of patients with more than six years of education. A report from the National Academy of Medicine clearly states that education is one of the most powerful social factors predicting health literacy [17]. Education level directly influences the foundational skills required for processing health information, such as reading, calculation, analysis, and reasoning, thereby affecting one’s ability to decode, comprehend, and apply complex health information. Therefore, patients with a lower education level may encounter comprehension barriers when interpreting drug instructions, complex treatment regimens, or health recommendations, leading to medication errors.

Meanwhile, this survey revealed that patients with urban resident basic medical insurance had significantly higher medication literacy scores (31.82 ± 11.21) compared to those covered by the NRCMS (24.88 ± 10.61) or those who were self-paying (22.33 ± 9.24). A robust healthcare security system not only directly alleviates financial burdens and reduces treatment interruptions due to cost constraints but also guides patients into more standardized and continuous healthcare service chains, helps establish stable doctor-patient relationships, and facilitates systematic health education.

Patients with a medical background or family members with medical backgrounds demonstrated significantly higher medication literacy scores. This aligns with the framework of health literacy systems [18], which posits that supportive physical and social environments enhance individuals’ capacity to adopt health behaviors. When individuals or their family members have a medical background, it establishes a health knowledge base within the family. This can provide immediate and reliable professional advice for daily health management, effectively compensating for the limited accessibility of primary healthcare services. Furthermore, the level of social support was positively correlated with medication literacy, consistent with the findings of Perngmark et al. [19]. This indicates that effective external supervision and support can enhance medication literacy among patients with hypertension. Patients with high levels of social support may receive more emotional comfort, medication supervision, and tangible support such as financial assistance, medical accompaniment, information sharing, and assistive tools, thereby stimulating their intrinsic motivation for disease management [20,21].

Pearson correlation analysis and multiple linear regression analyses conclusively demonstrated a strong positive correlation (r = 0.531, p < 0.001) between medication literacy and self-management ability. This profoundly reveals the dialectical unity of knowledge and practice in hypertension management [10]. Medication literacy was positively correlated with self-management, indicating that better medication knowledge and skills enhance overall self-care behaviors. Medication literacy provides the theoretical basis for active self-management, while regular self-monitoring and lifestyle modification reinforce appropriate medication use, thereby forming a virtuous cycle.

Furthermore, one of the core findings of this study is that medication literacy is a significant factor in achieving BP control targets (OR: 1.039, p = 0.021). After controlling for other variables, a 1-point increase in medication literacy was associated with an approximately 3.9% higher likelihood of achieving BP control targets. In-depth analysis revealed that among the four dimensions of medication literacy, the intergroup difference in medication knowledge was the most significant. This finding supports the importance of targeted health education to improve core medication knowledge and is consistent with previous research findings [10,14,18]. Health education is an effective approach to improving medication literacy, and culturally tailored health education can motivate patients to better manage their medication self-care [22]. 

Based on the above findings, we propose the following strategic considerations for hypertension prevention and control in Huayuan County and other ethnic minority regions with similar socioeconomic backgrounds.

Firstly, implement precise screening and stratified education. Routine and rapid medication literacy assessments should be carried out in primary healthcare institutions to identify high-risk populations. Health education for patients with low education levels, no stable health insurance, no medical background, and weak social support should be less academic and more context-based. For such populations, it is advisable to develop culturally appropriate health education materials using traditional methods such as oral education, written materials, and expert lectures. It is also important to explore more diverse and accessible approaches to integrate health education into local customs. The visual dialogue tool diagram developed by He et al. deconstructs abstract terms into images, integrating health education with audiovisual multi-sensory approaches to make health education more vivid and understandable [23]. The European Society of Cardiology recommends that patients participate in peer support groups [24]. Peer support [25] and family doctor contract services [26] have proven to be effective.

Secondly, establish a support network involving both families and communities. Family-based interventions can more effectively enhance the long-term health outcomes of patients with chronic diseases [27]. In multi-ethnic regions with well-established community traditions, such as Huayuan County, close-knit family and neighborhood networks not only serve as carriers of cultural inheritance but also as organized and functional health resources. The WHO advocates supporting and empowering family members as one of the core strategies to improve the quality of elderly healthcare [28]. Family health management training can be conducted to train the primary caregivers of patients, usually spouses or children, to serve as family medication supervisors. These supervisors directly participate in patients’ medication management by reminding them to take medications, assisting with medication dispensing, and recording medication intake. This helps reduce the incidence of missed or incorrect medication administration. This family-centered supervision mechanism has been proven to effectively improve treatment adherence among elderly patients with limited education or memory impairment [29]. Community health mutual-aid groups can be established to fully utilize peer support in emotional companionship, role-model guidance, and confidence building, systematically enhancing the role of social support in hypertension prevention and control. This model is sustainable in resource-limited regions.

Thirdly, explore supplementary pathways of digital technology empowerment. Remote and mountainous regions face challenges such as inconvenient transportation, scattered patient populations, difficulties in conducting in-person follow-ups, and high costs. By leveraging new media platforms and digital technologies, such as WeChat public accounts, short videos, and health-related apps, a regionally integrated management platform [30] with intelligent interactivity can be established to accomplish personalized risk assessment, medication reminders, BP trend feedback, and behavioral incentives. This effectively compensates for the limitations of traditional offline single-line education, offering low-cost, accessible, and continuous support. However, the design must take into account the operational ability and digital literacy of older users. The interface should be simple. Elderly individuals may require assistance from their children or guidance from primary care physicians.

This study has certain limitations. Firstly, the cross-sectional design cannot determine the causal relationship between medication literacy and BP control, only revealing associations. Future longitudinal studies or randomized controlled trials are needed to verify whether interventions in medication literacy directly lead to BP improvement. Secondly, the sample was derived from outpatient patients at a county-level hospital, which may introduce selection bias and fail to cover the most vulnerable populations who never seek medical care or who only obtain medications from village clinics. Future surveys should extend to rural areas to obtain more representative samples. Thirdly, using a standardized blood pressure measurement method may reduce errors caused by heterogeneity in measurement techniques. In the future, integrating electronic pill-counting, home BP monitoring, ambulatory BP monitoring, and smart wearable devices [30] would make the data more objective and reliable.

## Conclusions

The medication literacy level and BP control rate among hypertension patients in Huayuan County require improvement. Medication literacy is a crucial factor in promoting BP control. Implementing a medication literacy enhancement program for patients will contribute to BP control. Special attention should be paid to patient groups with lower education levels, no medical insurance, a lack of medical background, poor self-management ability, and insufficient social support. For hypertensive patients in multi-ethnic and remote areas, there is an urgent need to implement precision screening and stratified education and establish a support network for families and communities. Digital technology is expected to overcome the geographical and temporal constraints of traditional education.
